# Clinicopathological Characteristics and Prognostic Profiles of Breast Carcinoma with Neuroendocrine Features

**DOI:** 10.3390/life13020532

**Published:** 2023-02-15

**Authors:** Yue Qiu, Yongjing Dai, Li Zhu, Xiaopeng Hao, Liping Zhang, Baoshi Bao, Yuhui Chen, Jiandong Wang

**Affiliations:** 1 Graduate School, Chinese PLA Medical School, Beijing 100853, China; 2 Department of General Surgery, The First Medical Center, Chinese PLA General Hospital, 28 Fuxing Road, Haidian District, Beijing 100853, China; 3 Graduate School, Inner Mongolia Medical University, Hohhot 010110, China

**Keywords:** neuroendocrine tumors, breast neoplasms, prognosis

## Abstract

Background: Breast carcinoma with neuroendocrine features includes neuroendocrine neoplasm of the breast and invasive breast cancer with neuroendocrine differentiation. This study aimed to investigate the clinicopathological features and prognosis of this disease according to the fifth edition of the World Health Organization classification of breast tumors. Materials and Methods: A total of 87 patients with breast carcinoma with neuroendocrine features treated in the First Medical Center, Chinese PLA General Hospital from January 2001 to January 2022 were retrospectively enrolled in this study. Results: More than half of the patients were postmenopausal patients, especially those with neuroendocrine neoplasm (62.96%). There were more patients with human epidermal growth factor receptor 2 negative and hormone receptor positive tumors, and most of them were Luminal B type (71.26%). The multivariate analysis showed that diabetes and stage IV disease were related to the progression-free survival of breast carcinoma with neuroendocrine features patients (*p* = 0.004 and *p* < 0.001, respectively). Conclusion: Breast carcinoma with neuroendocrine features tended to be human epidermal growth factor receptor 2 negative and hormone receptor positive tumors, most of them were Luminal B type, and the related factors of progression-free survival were diabetes and stage IV disease.

## 1. Introduction

Breast carcinoma with neuroendocrine features consists of a group of diseases with high heterogeneity. It was reported that the incidence rate of breast carcinoma with neuroendocrine features ranged from 0.1% to 20% [1,2,3]. Breast carcinoma with neuroendocrine features included neuroendocrine neoplasm (NEN) of the breast and invasive breast cancer (IBC) with neuroendocrine differentiation. According to the latest World Health Organization (WHO) classification of breast tumors, NEN was divided into neuroendocrine tumor (NET) and neuroendocrine carcinoma (NEC) based on the degree of differentiation [3]. IBC with neuroendocrine differentiation was classed into breast carcinoma of no special type and breast carcinoma of special types, such as solid papillary carcinoma and the hypercellular subtype of mucinous carcinoma.

Since the third edition of the WHO classification of breast tumors was published, the definition and classification of this disease had changed greatly in different editions. As a result, there have been controversies surrounding the definition and classification of breast carcinoma with neuroendocrine features. The diagnostic criteria for subjects included in existing studies were not identical. As well, the research results were not completely consistent or were even contradictory [4,5]. Further, due to the rarity of the disease, few studies had been conducted, and those which have were mainly case reports [6,7,8]. At present, the treatment strategy of IBC of no special type is used directly in breast carcinoma with neuroendocrine features. The general practice guidelines for breast carcinoma with neuroendocrine features are still not formed. The TNM stage of breast carcinoma with neuroendocrine features was defined by the eighth version of the America joint committee on cancer staging systems [9]. According to the guidelines of the Chinese Society of Clinical Oncology published in 2020 [10], the minimum positive threshold of estrogen receptor (ER), progesterone receptor (PR), and Ki-67 are 1%, 1%, and 14%, respectively, and human epidermal growth factor receptor 2 (Her-2) (3+) or ISH positivity meant Her-2 positivity. Breast carcinoma with neuroendocrine features was divided into Luminal A (ER/PR positive, Her-2 negative with low Ki-67 index) disease, Luminal B (ER/PR positive, Her-2 negative with high Ki-67 index, or ER/PR positive, Her-2 positive) disease, Her-2 positive (ER and PR negative, Her-2 positive) disease, and Triple-negative (ER, PR, and Her-2 negative) disease according to molecular subtyping. To investigate the clinicopathological features and prognosis of this disease under the fifth edition of the WHO classification of breast tumors, we designed this study.

## 2. Materials and Methods

### 2.1. Study Groups

The data of 87 patients with breast carcinoma with neuroendocrine features treated in the First Medical Center, Chinese PLA General Hospital from January 2001 to January 2022 were retrospectively collected. Patients with breast carcinoma derived from other organs were excluded. Pregnant patients and patients who were breastfeeding were excluded as well. There were no patients without definite pathological diagnosis or without complete medical records. All procedures performed in this study involving human participants were in accordance with the Declaration of Helsinki (as revised in 2013). The study was approved by the ethics committee of the Chinese PLA General Hospital (NO.: S2022-746). Individual consent for this retrospective analysis was waived.

### 2.2. Study Variables

General information on the patients was collected, such as age at diagnosis, gender, laterality, smoking history, drinking history, body mass index (BMI), family history, menopause status, hypertension, diabetes, hyperlipidemia, T stage, Her-2 status, Ki-67, ER, PR, molecular typing, vessel carcinoma embolus, N stage, skin or chest wall invasion, distant metastasis, and stage.

Unique clinical features of the patients were analyzed, including clinical symptoms and history of thyroid diseases. Pathological characteristics of the patients were also described, such as detailed classification, expression of neuroendocrine markers, and ductal carcinoma in situ composition. The treatment strategy of the patients, such as neoadjuvant chemotherapy, surgery, and adjuvant therapy, was discussed. All patients enrolled in this study were followed up. The 5-year overall survival (OS), 5-year progression-free survival (PFS), and 5-year disease-specific survival (DSS) of patients in the study group were described. Finally, the factors related to 5-year PFS were analyzed.

### 2.3. Statistical Analysis

All statistical analyses of this study were performed using Stata Statistical Software version 15.1 (StataCorp LLC, College Station, TX, USA). The measurement data were described by median (inter-quartile range, IQR). Frequency was used to show the counting data. Comparison of counting data between two groups was conducted by Pearson chi-square test. Kruskal–Wallis H test was used to examine multiple comparisons of ranked counting data between groups. Kaplan–Meier method was used in survival analysis, and Log-rank test was used to compare different survival curves. Univariate and multivariate analysis were performed using Cox model. A two-tailed *p* < 0.05 was considered statistically significant, and all confidence intervals (CI) were expressed at 95% confidence level.

## 3. Results

### 3.1. General Clinicopathological Characteristics

The median age at diagnosis of the patients in the study group was 53 (42–64) years old. More than half of the patients were postmenopausal patients, especially those with neuroendocrine neoplasm (62.96%). The proportion of patients with Her-2 negative and hormone receptor (HR) positive tumors was high, and most of them were Luminal B type (71.26% vs. 28.74%). Around 49.43% of the patients had stage II disease. There was no difference between the NEN group and IBC with neuroendocrine differentiation group except in diabetes when analyzing general characteristics (*p* = 0.039) (Table 1).

### 3.2. Special Clinicopathological Features of Breast Carcinoma with Neuroendocrine Features Patients

A total of 22.99% of patients had clinical symptoms such as pain, nipple discharge, or both. About 21.84% of patients were complicated, with thyroid diseases such as thyroid nodule, diffuse thyroid disease, and thyroid cancer. A total of 34 patients with lymph node metastasis all had axillary lymph node metastasis, two cases also had supraclavicular lymph node metastasis, and one case had subclavian lymph node metastasis at the same time (Table 2).

**Figure 1:**
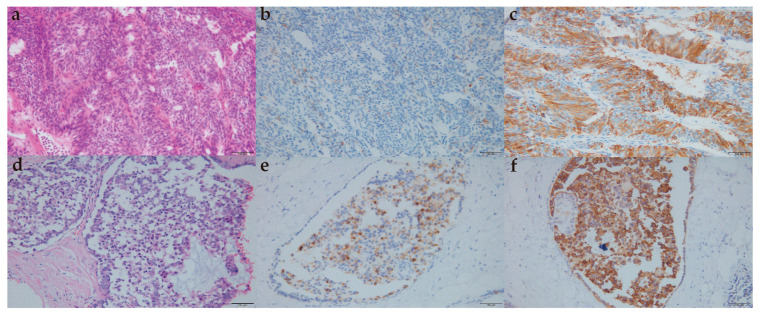
Morphological and immunohistochemical features. (**a**). H and E staining of NEC of the breast (×200); (**b**) positive immunostaining for chromogranin A of NEC of the breast (×200); (**c**) positive immunostaining for synaptophysin of NEC of the breast (×200); (**d**) H and E staining of no special type IBC with neuroendocrine differentiation (×200); (**e**) positive immunostaining for chromogranin A of no special type IBC with neuroendocrine differentiation (×200); (**f**) positive immunostaining for synaptophysin of no special type IBC with neuroendocrine differentiation (×200). NEC: neuroendocrine carcinoma; IBC: invasive breast cancer; H and E staining: Hematoxylin and Eosin staining.

A total of 87 cases with breast carcinoma with neuroendocrine features were classified into 29 cases of NEN and 58 cases of IBC with neuroendocrine differentiation (Table 3). After H and E staining and immunohistochemical staining, some tumor cells showed typical positive synaptophysin (Syn) and Chromogranin (CgA) staining (Figure 1). There were also some cases that had CD56+ tumors and neuron-specific enolase (NSE)+ tumors (Table 3).

### 3.3. Treatment and Follow-Up of Breast Carcinoma with Neuroendocrine Features Patients

Only 7 out of 87 patients received neoadjuvant chemotherapy. All patients received surgical treatment of the breast and/or axillary lymph node. There were 75 cases that underwent mastectomy, while the rest of the patients underwent breast-conserving surgery or nipple-areola complex-sparing mastectomy. As for adjuvant therapy, 81.61% of patients received chemotherapy, 31.03% of patients received radiotherapy, 73.56% of patients received endocrine therapy, and only 9.20% of patients received targeted therapy after surgery (Table 4).

A total of 87 patients were followed up, and 2 patients dropped out. The median follow-up time was 57 (25–74) months. During the follow-up period, local recurrence involving the breast, axilla, or chest wall occurred in five cases. There were 12 cases recorded of distant metastasis including bone, lung, liver, brain, retroperitoneal lymph node, and contralateral axillary lymph node. Eight cases died, six of them died of breast cancer and two died of other causes (Table 5).

### 3.4. Kaplan-Meier Survival Analysis of Breast Carcinoma with Neuroendocrine Features Patients

**Figure 2:**
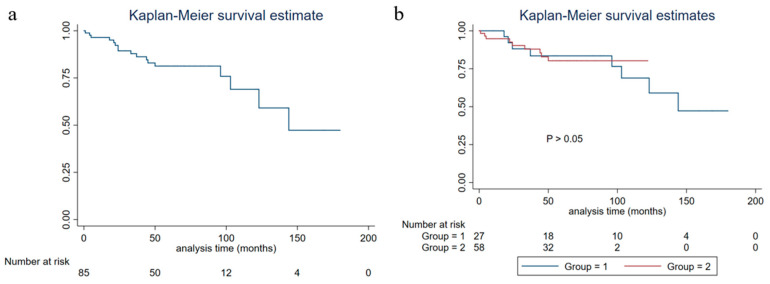
Progression-free survival of breast carcinoma with neuroendocrine features patients (group 1: neuroendocrine neoplasm patients; group 2: invasive breast cancer with neuroendocrine differentiation patients). (**a**) Progression-free survival of all breast carcinoma with neuroendocrine features patients; (**b**) progression-free survival of grouped breast carcinoma with neuroendocrine features patients.

**Figure 3:**
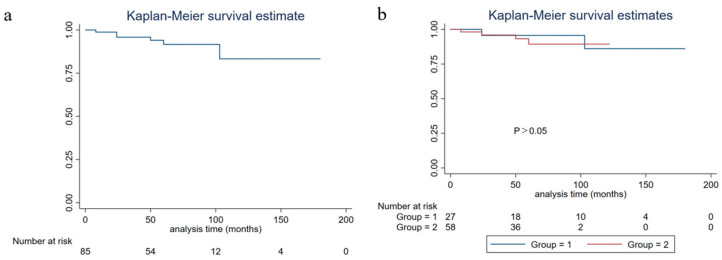
Disease-specific survival of breast carcinoma with neuroendocrine features patients (group 1: neuroendocrine neoplasm patients; group 2: invasive breast cancer with neuroendocrine differentiation patients). (**a**) Disease-specific survival of all breast carcinoma with neuroendocrine features patients; (**b**) disease-specific survival of grouped breast carcinoma with neuroendocrine features patients.

**Figure 4:**
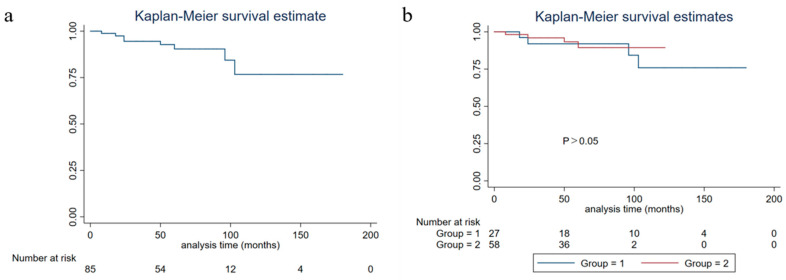
Overall survival of breast carcinoma with neuroendocrine features patients (group 1: neuroendocrine neoplasm patients; group 2: invasive breast cancer with neuroendocrine differentiation patients). (**a**) Overall survival of all breast carcinoma with neuroendocrine features patients; (**b**) overall survival of grouped breast carcinoma with neuroendocrine features patients.

The five-year PFS, five-year DSS, and five-year OS of all of the patients were 81.19% (95%CI: 0.6964–0.8869), 91.53% (95%CI: 0.8022–0.9651), and 90.25% (95%CI: 0.7901–0.9564), respectively. No significant differences were found in the five-year PFS, five-year DSS, and five-year OS between NEN and IBC with neuroendocrine differentiation (*p* > 0.05) (Figure 2, Figure 3 and Figure 4).

### 3.5. Univariate Analysis of PFS in Breast Carcinoma with Neuroendocrine Features Patients

The results of the univariate analysis of PFS showed that smoking history, diabetes, Her-2 positive disease, N3 stage disease, distant metastasis, and targeted therapy were related to the progression-free survival of breast carcinoma in neuroendocrine features patients (*p* = 0.038, *p* = 0.008, *p* = 0.003, *p* < 0.001, *p* < 0.001; *p* = 0.004, respectively). The results of Her-2 status and family history slightly missed the margin of significance (*p* = 0.097 and *p* = 0.092, respectively) (Table 6).

### 3.6. Multivariate Analysis of PFS in Breast Carcinoma with Neuroendocrine Features Patients

The results of the multivariate analysis of PFS showed that diabetes and stage IV disease were related to the progression-free survival of breast carcinoma in neuroendocrine features patients (*p* = 0.004 and *p* < 0.001, respectively). The result of targeted therapy showed a barely detectable statistical significance (*p* = 0.051) (Table 7).

## 4. Discussion

Breast carcinoma with neuroendocrine features is a group of heterogeneous tumors. Its definitions and diagnostic criteria have varied with the revisions of the WHO classification of breast tumors. As a result, the results of some studies on the clinicopathological characteristics of this disease have been controversial for a long time. Breast carcinoma with neuroendocrine features is a group of tumors that exhibit morphological features similar to those of neuroendocrine tumors of the gastrointestinal tract and lung [11]. Before 2003, there were no criteria for the definition and diagnosis of this disease. With the further study of breast carcinoma, the consensus on breast carcinoma with neuroendocrine features has gradually been formed. The third version of the WHO criteria of breast tumors defined it as >50% tumor cells with neuroendocrine differentiation confirmed by immunohistochemical staining [12]. From then on, it was recognized as single breast carcinoma entities named “neuroendocrine breast carcinomas”. In 2012, the WHO classification used the category of “carcinoma with neuroendocrine features” and described this disease as tumors expressing neuroendocrine markers to any extent [13]. It included well-differentiated NET, poorly differentiated NEC, and carcinoma with neuroendocrine differentiation. In this version, small cell neuroendocrine carcinoma (SCNEC) was brought into the NEC group. The current WHO classification adopted the term “NEN”, including well-differentiated (NET) and poorly-differentiated (NEC) tumors with predominant neuroendocrine differentiation [3]. The main distinction between the latest classification and the past version is that carcinoma with neuroendocrine differentiation without distinct or uniform enough neuroendocrine histological features and neuroendocrine marker expression is no longer classified as NET or NEC. In this version, large cell neuroendocrine carcinoma (LCNEC) was classified into NEC as well. All the criteria mentioned above are used for the classification of primary breast carcinoma with neuroendocrine features; however, before a diagnosis of primary NEN is made, the possibility of metastasis from other organs should be carefully ruled out. Immunohistochemistry staining is conducive to distinction between NEN derived from other organs from invasive mammary carcinoma with neuroendocrine features [14]. This study only discussed primary breast carcinoma with neuroendocrine features.

The results of the previous studies in different periods were different or even contradictory. Previous studies found that most patients were 50 years old or older [15,16], and that the clinical symptoms of this disease were mainly bloody nipple discharge [16], which was consistent with the results of this study. NEN can be divided into functional and non-functional tumors according to whether the tumor has hormone activity, and most NENs are non-functional. Functional NEN produces excessive hormones, leading to clinical symptoms such as diarrhea and facial flushing. Non-functional NENs do not produce enough hormones to cause these symptoms [17]. Paraneoplastic endocrine syndrome may occur in breast cancer with or without neuroendocrine differentiation [18]. There were few reports on paraneoplastic endocrine syndrome related to breast neuroendocrine tumors. One case with hyperprolactinemia was reported in the previous literature [19]. Studies have illustrated that patients often had ER/PR positive and Her-2 negative tumors [5,20], supporting this study. Another study found that neuroendocrine differentiation was more common in Luminal B breast cancer [21], which is consistent with the results of this study. However, among them, Her-2 positive patients were rare, only occasionally seen in case reports [22]. Research showed that breast carcinoma with neuroendocrine features was highly aggressive, with a high rate of local recurrence and distant metastasis, and a poor prognosis [5]. A study has also shown that its general clinical characteristics are not different from other breast cancers, and its biological behavior was not aggressive; on the contrary, it tended to be an independent good-prognosis subgroup [20].

Morphologically, the typical features of the lung/gastrointestinal tract NET, such as ribbons, cords, and rosettes, are not prominent in the breast NET; histological and immunohistochemical features of breast NEC are sometimes difficult to distinguish from lung NEC features [23]. CgA, Syn, NSE, and CD56 were neuroendocrine differentiation markers for breast carcinoma with neuroendocrine features [4]. It has been reported that only 23% of patients were detected with Syn+ and CgA+ at the same time [15]. The expression level of neuroendocrine markers in tumor tissues of patients in this study was also not high. There were significant differences in cytological characteristics between focal and diffuse neuroendocrine differentiated breast carcinomas [24]. However, when breast cancer of no special type with focal neuroendocrine differentiation was regarded as a separate entity, focal neuroendocrine differentiation had no obvious significance for its prognosis [15,25]. In this study, there was no difference between NEN and IBC with neuroendocrine differentiation in five-year OS, five-year DSS, and five-year PFS.

SCNEC was classified into NEC in 2012 and LCNEC was brought into NEC in 2019. A study indicated that approximately half of these patients had triple-negative breast cancer, with a 61.6% five-year DSS rate and 47.7% five-year OS rate [26]. Chemotherapy, surgery, and stage were predictive factors of prognosis. In this study, there was only one case of pure SCNEC and pure LCNEC, respectively, accounting for a relatively low proportion. The special type of breast cancer with neuroendocrine differentiation was rare. According to one study, half of the invasive solid papillary carcinomas were accompanied by neuroendocrine differentiation [7]. In this study, there were four cases of a special type of breast cancer with neuroendocrine differentiation, including two cases of solid papillary carcinoma, one case of invasive papillary carcinoma, and one case of type B mucinous carcinoma.

A study reported that the five-year OS and disease-free survival rates of HR positive/Her-2 negative breast cancer were 93.0% and 92.6%, respectively [27]. The PFS of breast carcinoma with neuroendocrine features was lower than that of the same molecular typing of breast cancer of no special type [28], which was consistent with the results of this study. The overall five-year PFS of patients in this study was 82.37% (95%CI: 0.7084–0.8966), five-year DSS was 91.53% (95%CI: 0.8022–0.9651), and five-year OS was 90.25% (95%CI: 0.7901–0.9564). The study of neoadjuvant therapy for this disease has been limited. In this study, there were seven patients receiving neoadjuvant chemotherapy, and none of them reached a pathological complete response. A study found that endocrine therapy or radiotherapy might improve the prognosis [5]. It was suggested that HR positive NEN patients receive endocrine therapy, especially those with SCNEC with recurrence and metastasis [29]. Endocrine therapy was also found to be effective for liver metastasis of breast cancer with neuroendocrine differentiation [6]. However, surgery was still the main treatment method, and the effect of chemotherapy on prognosis was still uncertain [16].

This study also analyzed the related factors of PFS. A study found that the prognostic factors of NEN of the breast were similar to those of gastrointestinal tract tumors [30], among which lymph node metastasis was an adverse factor of OS [5]. A previous study found that histological grade, pathological stage, ER status, and HER2 status were independent prognostic indicators of OS and disease-free survival [31]. The results of this study showed that diabetes and stage IV disease were related to the PFS of breast carcinoma with neuroendocrine features patients. The influence of diabetes on the PFS of this disease may be related to higher BMI, and there is a lack of relevant research at present.

### Limitations

This study was a retrospective study, and the sample size was relatively insufficient. We failed to compare this disease with other types of breast cancer because of lacking a control group. In addition, this study did not describe its imaging characteristics. Because of the rarity of this disease, there was a gap between the length of follow-up time of the patients.

## 5. Conclusions

Breast carcinoma with neuroendocrine features is relatively rare compared with other types of breast cancer. In this study, it was illustrated that this disease tended to be HR+/Her-2- tumor. In addition, diabetes and stage IV were related to the PFS of patients. These results may provide evidence for the treatment and prognosis prediction of breast carcinoma with neuroendocrine features. Further studies such as large-sample randomized clinical trials are needed to validate the theoretical value and practical significance of these findings and improve understanding of this disease.

## Figures and Tables

**Table 1:** General clinicopathological characteristics of breast carcinoma with neuroendocrine features patients.

Category	Breast Carcinoma with Neuroendocrine Features (n = 87) (%)	NEN Group	IBC with Neuroendocrine Differentiation (n = 58) (%)	*p* Value
		(n = 29) (%)		
Age at diagnosis (years)				0.875
≤60	55 (63.22%)	18 (62.07%)	37 (63.79%)	
>60	32 (36.78%)	11 (37.93%)	21 (36.21%)	
Gender				0.109
Female	85 (97.7%)	27 (93.10%)	58 (100.00%)	
Male	2 (2.30%)	2 (2.30%)	0 (0.00%)	
Laterality				0.129
Left	44 (50.57%)	18 (62.07%)	26 (44.83%)	
Right	43 (49.43%)	11 (37.93%)	32 (55.17%)	
Smoking history				1
Yes	2 (2.30%)	1 (3.45%)	1 (1.72%)	
No	85 (97.70%)	28 (96.55%)	57 (98.28%)	
Drinking history				0.333
Yes	1 (1.15%)	1 (3.45%)	0 (0.00%)	
No	86 (98.85%)	28 (96.55%)	58 (100.00%)	
BMI (kg/m^2^)				0.128
≥24	47 (54.02%)	19 (65.52%)	28 (48.28%)	
<24	40 (45.98%)	10 (34.48%)	30 (51.72%)	
Family history				1
Yes	1 (1.15%)	0 (0.00%)	1 (1.72%)	
No	86 (98.85%)	29 (100.00%)	57 (98.28%)	
Menopausal status ^a^				0.332
Yes	47 (55.29%)	17 (62.96%)	30 (51.72%)	
No	38 (44.71%)	10 (37.04%)	28 (48.28%)	
Hypertension				0.492
Yes	23 (26.44%)	9 (31.03%)	14 (24.14%)	
No	64 (73.56%)	20 (68.97%)	44 (75.86%)	
Diabetes				0.039
Yes	7 (8.05%)	5 (17.24%)	2 (3.45%)	
No	80 (91.95%)	24 (82.76%)	56 (96.55%)	
Hyperlipidemia				0.368
Yes	20 (22.99%)	5 (17.24%)	15 (25.86%)	
No	67 (77.01%)	24 (82.76%)	43 (74.14%)	
T stage (AJCC 8th)				0.2
1	45 (51.72%)	18 (62.07%)	27 (46.55%)	
2	35 (40.23%)	9 (31.03%)	26 (44.83%)	
3	7 (8.05%)	2 (6.90%)	5 (8.62%)	
4	0 (0.00%)	0 (0.00%)	0 (0.00%)	
Her-2 status				0.323
Positive	11 (12.64%)	2 (6.90%)	9 (15.52%)	
Negative	76 (87.36%)	27 (93.10%)	49 (84.48%)	
Ki-67				0.229
Positive	72 (82.76%)	22 (75.86%)	50 (86.21%)	
Negative	15 (17.24%)	7 (24.14%)	8 (13.79%)	
ER				0.857
Positive	67 (77.01%)	22 (75.86%)	45 (77.59%)	
Negative	20 (22.99%)	7 (24.14%)	13 (22.41%)	
PR				0.434
Positive	71 (81.61%)	25 (86.21%)	46 (79.31%)	
Negative	16 (18.39%)	4 (13.79%)	12 (20.69%)	
Molecular typing (CSCO 2020)				0.494
Luminal A	13 (14.94%)	5 (17.24%)	8 (13.79%)	
Luminal B	62 (71.26%)	21 (72.41%)	41 (70.69%)	
Her-2 positive	1 (1.15%)	0 (0.00%)	1 (1.72%)	
Triple negative	11 (12.64%)	3 (10.34%)	8 (13.79%)	
Vessel carcinoma embolus				0.702
Yes	17 (19.54%)	5 (17.24%)	12 (20.69%)	
No	70 (80.46%)	24 (82.76%)	46 (79.31%)	
N stage (AJCC 8th)				0.549
0	53 (60.92%)	19 (65.52%)	34 (58.62%)	
1	18 (20.69%)	5 (17.24%)	13 (22.41%)	
2	14 (16.09%)	5 (17.24%)	9 (15.52%)	
3	2 (2.30%)	0 (0.00%)	2 (3.45%)	
Skin or chest wall invasion				---
Yes	0 (0.00%)	0 (0.00%)	0 (0.00%)	
No	87 (100.00%)	29 (100.00%)	58 (100.00%)	
Distant metastasis				0.55
Yes	2 (2.30%)	0 (0.00%)	2 (3.45%)	
No	85 (97.70%)	29 (100.00%)	56 (96.55%)	
Stage (AJCC 8th)				0.577
I	26 (29.88%)	11 (37.93%)	15 (25.86%)	
II	43 (49.43%)	11 (37.93%)	32 (55.17%)	
III	16 (18.39%)	7 (24.14%)	9 (15.52%)	
IV	2 (2.30%)	0 (0.00%)	2 (3.45%)	

BMI: body mass index; ER: estrogen receptor; PR: progesterone receptor. Her-2: human epidermal growth factor receptor 2; IBC: invasive breast cancer; AJCC 8th: The eighth version of America joint committee on cancer staging system; CSCO 2020: The guidelines of Chinese Society of Clinical Oncology in 2020. ^a^ There were two male breast cancer patients in the study group.

**Table 2:** Supplement of part clinical features of breast carcinoma with neuroendocrine features patients.

Category	n = 87
Clinical symptoms	
Yes	20 (22.99%)
Pain	14
Nipple discharge	5
Nipple discharge with pain	1
Paraneoplastic symptoms	0
No	67 (77.01%)
Thyroid diseases	
Yes	19 (21.84%)
Thyroid nodule	15
Diffuse thyroid disease	3
Thyroid cancer	1
No	68 (78.16%)
Lymph node metastasis	
Yes	34 (39.08%) ^a^
Axillary lymph node metastasis	34
Supraclavicular lymph node metastasis	2
Subclavian lymph node metastasis	1
No	53 (60.92%)

^a^ One patient had axillary lymph node metastasis, supraclavicular lymph node metastasis, and subclavian lymph node metastasis at the same time. One patient had axillary lymph node metastasis and supraclavicular lymph node metastasis at the same time.

**Table 3:** Supplement of part pathological characteristics of breast carcinoma with neuroendocrine features patients.

Category	n = 87
Classification	
NEN	29 (33.34%)
NET	26
Simple type	21
Mixed type	5
NEC	3
Small cell carcinoma	1
Large cell carcinoma	1
Mixed type	1
IBC with neuroendocrine differentiation	58 (66.66%)
IBC of no special type	
Invasive ductal carcinoma	50
IBC of special type	
Solid papillary carcinoma	2
Invasive papillary carcinoma	1
Type B mucinous carcinoma	1
Mixed type	4
Neuroendocrine markers	
Syn	
0	3 (3.45%)
1+	76 (87.35%)
2+	4 (4.60%)
3+	4 (4.60%)
CgA	
0	10 (11.49%)
1+	11 (12.64%)
2+	25 (28.74%)
3+	2 (2.30%)
NK	39 (44.83%)
CD56	
0	11 (12.64%)
1+	26 (29.88%)
2+	42 (48.28%)
3+	1 (1.15%)
NK	7 (8.05%)
NSE	
0	1 (1.15%)
1+	7 (8.05%)
2+	2 (2.30%)
3+	1 (1.15%)
NK	76 (87.35%)
Ductal carcinoma in situ composition	
Yes	29 (33.33%)
No	58 (66.67%)

NEN: neuroendocrine neoplasm; NET: neuroendocrine tumor; NEC: neuroendocrine carcinoma; IBC: invasive breast cancer; Syn: synaptophysin; CgA: chromogranin A; NSE: neuron-specific enolase; NK: not known.

**Table 4:** Treatment strategy of breast carcinoma with neuroendocrine features patients.

Category	n = 87
Neoadjuvant chemotherapy	
Yes	7 (8.05%)
No	80 (91.95%)
Surgery treatment	
Breast	
Mastectomy	75 (86.20%)
BCS	8 (9.20%)
NSM	3 (3.45%)
None ^a^	1 (1.15%)
Axillary lymph node	
SLNB	19 (21.84%)
ALND	67 (77.01%)
None ^b^	1 (1.15%)
Adjuvant therapy	
Chemotherapy	
Yes	71 (81.61%)
No	16 (18.39%)
Radiotherapy	
Yes	27 (31.03%)
No	60 (68.97%)
Endocrine therapy	
Yes	64 (73.56%)
No	23 (26.44)
Targeted therapy	
Yes	8 (9.20%)
No	79 (90.80%)

BCS: breast conserving surgery; NSM: nipple-areola complex sparing mastectomy; SLNB: sentinel lymph node biopsy; ALND: axillary lymph node dissection. ^a^ One patient had occult breast cancer, receiving ALND only. ^b^ One patient who was elderly received breast surgery only.

**Table 5:** Local recurrence, distant metastasis, and mortality of breast carcinoma with neuroendocrine features patients.

Category	n = 85 ^a^
Local recurrence	5 (5.88%)
Breast	1
Chest wall	3
Axilla	1
Distant metastasis	12 (14.14%)
Bone	4
Lung	6
Liver	2
Brain	3
Retroperitoneal lymph node	1
Contralateral axillary lymph node	2
Death	8 (9.41%)
Breast cancer	6
Other causes	2
Follow-up time (months, IQR)	57 (25–74)

IQR: inter-quartile range. ^a^ There were 87 patients totally, and two patients dropped out.

**Table 6:** Univariate analysis of PFS in breast carcinoma with neuroendocrine features patients.

Variable	HR (95% CI)	*p* Value
Age (year)		
>60	1	
≤60	0.9670 (0.3566–2.6224)	0.947
Gender		
Female	1	
Male	1.6943 (0.2138–13.4258)	0.618
Laterality		
Right	1	
Left	0.4458 (0.1587–1.2522)	0.125
Smoking history		
No	1	
Yes	9.1939 (1.1251–75.1266)	0.038
Drinking history		
No	1	
Yes	---	1
BMI (kg/m^2^)		
<24	1	
≥24	1.2394 (0.4535–3.3873)	0.676
Family history		
No	1	
Yes	5.8103 (0.7485–45.1052)	0.092
Menopausal status		
No	1	
Yes	0.7091 (0.2629–1.9125)	0.497
Hypertension		
No	1	
Yes	0.8888 (0.2882–2.7409)	0.837
Diabetes		
No	1	
Yes	4.8491 (1.5089–15.5837)	0.008
Hyperlipidemia		
No	1	
Yes	1.1692 (0.3771–3.6253)	0.787
T stage		
1	1	
2	1.6177 (0.5770–4.5348)	0.36
3	1.5048 (0.3172–7.1392)	0.607
Her-2 status		
Negative	1	
Positive	2.6226 (0.8390–8.1975)	0.097
Ki-67		
Negative	1	
Positive	1.1088 (0.3391–3.6256)	0.864
ER		
Positive	1	
Negative	1.6555 (0.5819–4.7100)	0.345
PR	1	
Positive	1.6112 (0.5242–4.9525)	
Negative		0.405
Molecular typing		
Luminal A	1	
Luminal B	1.7925 (0.3694–8.6984)	0.469
Her-2 positive	72.1912 (4.4462–1172.1380)	0.003
Triple negative	2.3484 (0.3846–14.3384)	0.355
Vessel carcinoma embolus		
No	1	
Yes	0.4462 (0.1013–1.9650)	0.286
N stage		
0	1	
1	1.6375 (0.5346–5.0153)	0.388
2	0.9315 (0.1976–4.3919)	0.929
3	150.1349 (12.6995–1774.9190)	<0.001
Skin or chest wall invasion		
No	1	
Yes	---	---
Distant metastasis		
No	1	
Yes	133.1195 (11.7712–1505.4410)	<0.001
Stage		
I	1	
II	2.4426 (0.6494–9.1871)	0.186
III	1.5589 (0.3132–7.7588)	0.588
IV	243.9231 (17.2429–3450.6020)	<0.001
Classification		
IBC with neuroendocrine differentiation	1	
NEN	0.9300 (0.3073–2.8146)	0.898
Neoadjuvant chemotherapy		
Yes	1	
No	0.4378 (0.1241–1.5447)	0.199
Breast treatment		
Mastectomy	1	
BCS	1.1105 (0.2508–4.9179)	0.89
NSM	1.1265 (0.1455–8.7217)	0.909
Axillary lymph node treatment		
ALND	1	
SLNB	0.6931 (0.1972–2.4355)	0.568
Chemotherapy		
Yes	1	
No	2.1270 (0.7460–6.0646)	0.158
Radiotherapy		
Yes	1	
No	1.1213 (0.4000–3.1428)	0.828
Endocrine therapy		
Yes	1	
No	1.4339 (0.5213–3.9437)	0.485
Targeted therapy		
Yes	1	
No	0.1838 (0.0579–0.5840)	0.004

BMI: body mass index; ER: estrogen receptor; PR: progesterone receptor. Her-2: human epidermal growth factor receptor 2; NEN: neuroendocrine neoplasm; IBC: invasive breast cancer; BCS: breast conserving surgery; NSM: nipple-areola complex sparing mastectomy; SLNB: sentinel lymph node biopsy; ALND: axillary lymph node dissection; PFS: progression-free survival; HR: hazard ratio; CI: confidence interval.

**Table 7:** Multivariate analysis of PFS in breast carcinoma in neuroendocrine features patients.

Variable	HR (95%CI)	*p* Value
Smoking history		
No	1	
Yes	---	1
Diabetes		
No	1	
Yes	7.2526 (1.8874–27.8691)	0.004
Molecular typing		
Luminal A	1	
Luminal B	0.8695 (0.1472–5.1356)	0.877
Her-2 positive	---	---
Triple negative	1.5148 (0.2420–9.4801)	0.657
Stage		
I	1	
II	1.9789 (0.5132–7.6316)	0.322
III	0.8796 (0.1515–5.1080)	0.886
IV	2.44 × 10^19^ (5.72 × 10^17^–1.04 × 10^21^)	<0.001
Targeted therapy		
Yes	1	
No	1.7777 (0.0313–1.0085)	0.051

PFS: progression-free survival; HR: hazard ratio; CI: confidence interval.

## Data Availability

The data that support the findings of this study are available from the corresponding authors, [J.W. and Y.C.], upon reasonable request.
